# Selections

**Published:** 1892-07

**Authors:** 


### *SELECTIONS.*

---

THE HEALING OF TUBERCULOSIS.—Prof. William Osler in a recent paper says: "I have carefully reviewed the records of 1,000 post mortems, dictated in all instances by myself, with reference to this question. In 216 cases death was caused by pulmonary tuberculosis. Excluding the simple fibroid puckering, the local thickening of the pleura, and the solitary caseous or calcareous mass, there was among the remaining 784 cases, 59 or 5.05 per cent. in which persons dying of other diseases presented undoubted tuberculous lesions in the lungs.

The observations upon this subject have been of late numerous, and the discrepancies in the figures are due largely to an absence of a uniform criterion as to what should be regarded as obsolete or quiescent tubercles. If the fibroid patches are to be included, as in some of the following statistics, the percentage is high. Heitler analyzed the Vienna post mortem records and found that in 16,562 cases, in which the death was not directly caused by phthisis, there were 780 instances of obsolete tubercle, a percentage of 4.7. He excluded, as I have done, the simple fibroid induration at the apex. With each decimal period, up to the sixtieth year, the number of cases increased.

In 27 per cent. in 400 bodies, Bollinger found evidence of healing of tubercular lesions in the lungs. Staudacher, in 787 cases, found apex cirrhosis in 202. Massini found evidences of healing in 39 per cent. in 228 bodies examined. Harris of Manchester has examined 200 bodies, keeping this object specially in view. Excluding the deaths from phthisis and persons under twenty, there were left 139 cases for analysis, in 54 of which there were relics of former active tuberculosis 38.84 per cent. The greater number of these were in the third, fourth and fifth decades. The large proportion here given is accounted for by the inclusion of the fibroid cicatrices as well as the caseous masses.

I heard the statement made in Paris that of the bodies examined in the morgue, the majority of which are suicides or persons accidentally killed, nearly seventy-five per cent. present evidences of old tuberculous lesions.

These facts demonstrate, first, the wide-spread prevalence of tuberculosis; and secondly, the fact as shown by my figures that at least one-fourth of all infected persons recover spontaneously. In the great majority of these cases the disease is very limited and has made no progress, and in many instances could not have given physical signs. But even in more advanced disease, where the local indications are marked and bacilli and elastic tissue present in the sputum, arrest is by no means infrequent, and although post mortem evidence shows that we are wrong in speaking of the process as *cured*, yet the condition is consistent with comparatively good health.

We may say then that in one-fourth of all persons infected the disease is never manifest, but remains local, and the lesions gradually heal In another fourth of those attacked, local signs develop, but the physiological resistance is sufficient to arrest the process, or in modern language the battle is against the invaders, the day is with the tissues, and a permanent time is agreed upon, or sometimes a permanent withdrawal of the enemy. The remaining fifty per cent. of those infected fight for months and years losing battles until the final defeat comes.—*Medical Progress.*

HERNIA, OPERABLE AND INOPERABLE.—Dr. Thos. H. Manley, in a paper on this subject, submitted at the late meeting of the American Medical Association, concluded as follows: Although, during the past fifteen or twenty years, very much has been written on hernial operations, much in America, but more in Europe, so far as can be learned from current literature, no one has yet made an attempt in a methodical manner, in any brochure or treatise published, to systematically describe and classify those herniæ which are operable and those which are not. For the purpose, of at least in part, filling in this void, this essay is offered.

In epitomizing the subject and considering the question of treatment of hernia, in a general way, it may be said with something approaching definite certainty:

1. That, inasmuch as no operative technique yet devised, or even can be, will always effectually remove the causes of any species of hernia, in consequence of this, a permanent, enduring cure is quite out of the question in certain cases.

2. The radical cure of hernia may be regarded as one of the most satisfactory operations in general surgery. The fact that the disease often relapses constitutes no valid objection against surgical intervention; for this is commonly the case in the major part of operation performed on the human body.

3. A radical operation for a non-strangulated hernia, which gives no serious inconvenience, is not a justifiable procedure, and should not be encouraged, unless there are pressing reasons for it; as the presence of a neoplastic formation in the sac, or this latter is occupied by an ovary, or some other viscus.

4. Hernial operations should not be performed in extremes of age. In early life there is seldom pressing necessity for them. In very advanced age the risk, immediate or remote to life or health, involved by operation more than compensates for the prospective benefit.

5. Unless there are specially contraindicating factors in given cases, inguinal hernia should always be treated by radical operation in women, which, with them is so often attended by a permanent cure.

6. As a rule, all operations on reducible or incarcerated herniæ are radically curative, though the disease commonly relapses; nevertheless, the immediate danger of strangulation has been removed, and the hernia has been placed in a position to be more comfortably borne by a truss.

7. Every operation for strangulated vaginal or femoral hernia should always include such additional steps, as will effect thereafter an obliteration of the canal without undue pressure on the tubular structures passing through it.

8. Very large, old herniæ, in any of the abdominal regions are not operable, except in the event of strangulation, as their sudden return is often attended with mortal consequences.—*Weekly Medical Review.*

---

ACUTE AND CHRONIC DYSENTERY.—Dr. P. B. Green, of Wytheville, thinks that for the most part dysentery should be treated as a septic disease by antiseptics, such as iodoform, salicylic acid, creasote, naphthalin, etc. Sodium salicylate was perhaps the one to be generally preferred.

Treatment by rectal injections and antiseptics had invariably proved successful in adults, and in most cases in children except infants. He preferred warm solutions of mercuric bichloride (from 1 to 10,000 to 1 to 3,000) every six to twelve hours. Listerine, salol, fluid extract of hydrastis, and the salicylates were other good antiseptics.

Dr. Bedford Brown, of Alexandria, suggests the following: Begin treatment with calomel as a cathartic, and follow this by a cleansing dose of Epsom salts. Then begin with antiseptic injections—about two drachms of carbolic acid to two or more pints of water. By the mouth, give a tablespoonful every three hours of—

℞. Aromatic sulphuric acid. ................ ʒij.

Sulphate of magnesium.................. ℥ss.

Deodorized tincture of opium............ ʒij.

Syrup of orange-peel...................... ℥j.

Peppermint water.......................... ℥iij.

Water........................................ ℥ij.

Occasionally full doses of Epsom salts were often needed to relieve congestions, correct secretions, etc. In severer cases, use more decided antiseptics, such as naphthalin, salol, and phenacetin, five grains of each every three hours, and irrigate the bowels twice daily with creolin. If adynamia occurred, marked by intenseiy fœtid discharges of blood mixed with mucus, pus, sloughs, etc., use injections of an ounce or two of hydrogen peroxide in a pint of water. In malignant hæmorrhagic forms, give half an ounce of turpentine in a pint of emulsion, and repeat the following every two or three hours:

| | | |
|---|---|---|
| ℞. | Opium.......................................... | gr. ss. |
| | Tannin.......................................... | gr. ii. |
| | Strychnine sulphate.................... | gr. $\frac{1}{05}$. |
| | Iodoform.......................................... | gr. j. |
| | Creasote.......................................... | gtt. j. |

M.

In chronic dysentery, the septic fæcal matter, by constantly passing over the raw or wounded surfaces, kept up the septic condition. Tepid-water irrigations, followed by half-gallon injections twice a day of solutions of hydrogen peroxide, creolin, or ichthyol, would do good. It was best to administer these irrigations through a soft-rubber tube, about fifteen inches long, passed well up into the colon.—*Med. and Surg. Reporter.*

---

THE SUMMER DIARRHŒA OF INFANTS.—Concerning the treatment of the summer complaint of infants Dr. Waugh says (*Medical World*): Whenever the child's stools begin to be offensive, there is trouble brewing. Then they need a little corrective, like this:

| | | |
|---|---|---|
| R. | Tincture hydrastis, . . | drams iv. |
| | Potassii carb., . . . . | dram j. |
| | Syrupi rhei aromat., . | ounces iijss. |

M. Sig.—Ounce j every four hours.

Many other remedies accomplish the same object; calomel, ipecac, and the intestinal antiseptics are all of value; but I prefer the above combination, as neutralizing acidity, stimulating the secretions of the digestive fluids and exerting a tonic influence on the whole system. If a few doses do not produce natural stools, I begin at once with the sulpho carbolate of zinc. To a child in its second summer I give from a quarter to one grain, every one to two hours; preferring small doses repeated frequently. The best formula is this:

℞. Zinci sulpho-carbolat, . . . grains iv.
Bismuth subnitrat, . . . . grains xv.
Testæ preparat, . . . . . grains xv.
M. et in chart. No. xij div.
S. One every hour.

Instead of the oyster shell I often give saccharated pepsin, when a digestant is indicated. If the symptoms continue I give more of the zinc. If the bowels become very loose, I give enemas of warm flax seed tea, with ten grains of the sulpho carbolate to four ounces. This is the standard treatment of the summer complaint of infants, in all its grades; the doses being given more frequently in the more serious cases. If the nausea and vomiting have ceased and the bowels continue loose, with dysenteric symptoms, give an enema of four ounces hot water with half a grain of nitrate of silver. For several years I have employed these remedial measures each summer, and have had no reason to change them.

---

TRIBUTE TO SOUTHERN SURGEONS.—The *Dublin Journal of Medical Sciences* for April, again discusses the comparative merits of ether and chloroform. Among other things, it attacks the statement made by Surgeon-Major Lawrie, that a majority of American surgeons prefer ether on account of its greater safety. The *Dublin Journal* is evidently on the side of chloroform. Passing by the main points of the discussion, we quote only the following:

" Like many others, Dr. Lawrie confounds the New England States with the United States. In so doing, he reminds us of those politicians who in describing Ireland leave out Ulster. The New England States are not the United States, neither is their opinion the American opinion. The men who raised American surgery to its present high standard were Southerners, men of the cotton States; and when the State's-rights War occurred, the Confederate surgeons exhibited a skill and resourcefulness, and produced better results, than (*sic*) up to that time any military surgeons ever recorded; and this was done amidst the greatest difficulties. The men who have this splendid record to their credit, without exception, are chloroformists. Two of the most distinguished of these Southern surgeons have expressed their views in no uncertain manner on the chloroform questions, as may be seen by reference to Mr. Foy's book, 'Anæsthetics, Ancient and Modern.'

"That the Eastern States advocate ether in season and out of season, and that they are intolerant of any difference of opinion, is notorious; but nevertheless, the United States refuses to be dominated by a noisy, didactic faction, and with the majority of American surgeons chloroform continues to be the favorite anæsthetic."

---

CHRONIC URETHRITIS.—Chronic Urethritis is treated by Dr. A. G. Gerster, of New York, by injections of a 1-2000 solution of potassium permanganate, carried to a point between the sphincter of the bladder and the cut off muscle.

A marked improvement usually quickly follows, and on the degree of improvement depends the frequency with which the applications should be made.

In very chronic cases it is necessary to resort to the deep injection of from three to five minims of a five per cent. solution of nitrate of silver, once, twice or three times a week according to the severity of the case.

These injections are made after the patient has urinated.

He is placed on his back and the injector introduced beyond the cut-off muscle into the neck of the bladder, but not into the bladder.

It is at this point that the trouble lies, between cut-off muscle and the vésical sphincter.—*Medical Mirror.*

THE TREATMENT OF GONORRHŒA.—My treatment of gonorrhœa in all stages has for long been very monotonous. Almost without regard to stage or degree of severity, I prescribe the same remedies. I have long ago laid aside the traditions of my student days, which taught that salines only should be used in the acute stages, and that abortive plans were dangerous. I always use abortive measures, and mostly, I believe, succeed. At any rate I never encounter ill consequences, and complications are rare. My prescription is a partnership of three different remedies and it is, I believe, important that they should all be used. First an injection of solution of chloride of zinc, two grains to the ounce; next, sandalwood oil capsules; and lastly, a purgative night dose with bromide of potassium. The injection is used three or four times a day, the capsules (ten or twenty minims) taken three times a day. The ingredients of the night dose are three drachms of Epsom salts and half a drachm of bromide of potassium. It is, I believe, the action of the last-named in preventing congestion of the parts which makes the abortive measures safe. Moderate purgation and entire abstinence from stimulants are essential. If the case is very acute and attended by swelling of the corpus spongiosum, I sometimes prescribe tartar emetic or tincture of aconite, but it is very seldom indeed that these are necessary. If the patient be well purged, there is no risk whatever in an abortive treatment from the day that he comes under treatment. The risk of orchitis, prostatitis, cystitis, etc., comes in cases which have been allowed to develop rather than in those treated abortively. I should as soon think of delaying to use local measures in gonorrhœa as I should in purulent ophthalmia.—*Jonathan Hutchinson in Archives of Surgery.*

---

THE USE OF DRUGS IN THE TREATMENT OF EARLY PHTHISIS.—This is the title of a paper read by Dr. J. C. Thorogood at the last annual meeting of the British Medical Association. For those patients who are unable to avail themselves of the benefits of a change of air and clime, the writer is of the opinion that a great deal can still be accomplished by drugs. In apical catarrh and the persistent consolidation following pneumonia, he lays great stress on the value of the hypophosphites, especially the hypophosphite of soda, in five grain doses in water or infusion of columba three times daily. Favorable results were thereby obtained in cases which had resisted other methods. Thickening of the pleural membranes also yields to this plan of treatment, but not when complicated with effusion.

A process of fatty change and liquefaction is supposed by Dr. Thorogood to be set up, and absorption follows. This latter effect in some cases is so active as to lead to a marked rise in temperature, when the dose must be diminished. Care must also be had in its administration in recurring hæmoptysis.

To check sweating and diarrhœa the calcium hypophosphite is recommended. The inhaling aspirator, used with iodoform dissolved in ether, alcohol or eucalyptus oil, is also considered as exerting a favorable influence.

Next to iodoform as an inhalation, is placed the best German creosote. The perforated zinc respirator containing either of the above drugs should be worn for an hour three times daily. Confidence is had also in persistent and even severe counter-irritation with croton liniment, or linimentum terebinthinæ.—*Medical Fortnightly.*

---

SPURIOUS SALOL.—It appears that recently some spurious mixtures have been retailed as pure salol. The pure article is found to be readily identified by placing a few drops of nitro-sulphuric acid in a watch-glass and adding a little salol powder. The mixture soon takes a yellow color and, on being stirred about with a glass rod, this color passes to brown, and then to green. As soon as this coloration has been noticed, the watch-glass and its contents are placed in a porcelain measure with about three quarters of an ounce of water, and the mixture shaken up. The liquid then takes a rose color, and the green color again appears if ammonia is added to the liquid. When resorcin is treated exactly in the same manner, the mixture becomes immediately of a very dark blue color. If then it is treated as above with water, a red color derived from resorcin, is produced, but by the addition of ammonia, it turns blue and not green, as is the case with salol.—*American Lancet.*

CALOMEL IN TYPHOID FEVER.—De Simone (*Rif. Med.*), is of opinion that, whereas during the first ten days of enteric fever the high temperature is due to systemic infection with the specific typhoid bacillus, after that period the fever is of a different type and is mainly due to secondary infection with other bacteria derived from the intestine, which find easy access to the tissues through the inflamed and ulcerated Peyer's patches. Having found in calomel an excellent intestinal disinfectant in epidemics of cholera and dysentery, he has tried this drug in cases of typhoid with very good results. He gives small doses (five centigrammes with one centigramme of opium) every two to four days. It has no influence on the fever of the first seven to ten days (that due to the presence of the B. typhosus in the tissues), but after this he has found that in many instances it completely cut short the secondary oscillations of temperature (probably by disinfectant action on the intestine).

De Simone therefore concludes that: (1) In calomel we possess an excellent intestinal antiseptic; (2) small doses are powerless to arrest the fever of the first period of typhoid, but completely cut short that of the later period; (3) they act in the case as energetic disinfectants of the typhoid ulcers and protect them from the pathogenic microbes of the intestine.—*Supp. British Med. Journal.*

---

PROPHYLAXIS OF SCARLATINOUS NEPHRITIS.—Dr. Ziegler (*La Semaine Medicale*, No. 4, 1892) puts his scarlatina patients upon a milk diet from the very first, and in over a hundred cases he has not seen a renal complication. During the first few days, when the anorexia is complete, the child is given a little milk diluted with mineral water. When the appetite returns the child is given from a pint to three quarts of milk a day for the first three weeks; the milk is first boiled before administering. Now and then the child may be permitted to eat a piece of bread or a biscuit. This is continued in all its strictness for the first three weeks of the disease, to return gradually to the ordinary food.—*Cin. Lancet Clinic.*

TREATMENT OF SOFT CHANCRES AND SUPPURATING BUBOES.—Dr. Gamel, of Marseilles, France, praises camphorated carbolic acid in soft chancres. It hinders the development of phagedena and renders ordinary (soft) chancres simple wounds, to cicatrize in three days. Very extensive chancrous wounds healed rapidly under its influence. He employs the following formula:

R. Crystallized carbolic acid, gms. 10

(ʒijss).

Camphor, . . . gms. 25

(ʒvi).

Mix and warm on a water bath.

This forms a limpid, syrupy liquid, similar to glycerine, and of a very agreeable odor. Th e dressings should be repeated twice a day. Clean the sore well with absorbent cotton and apply small pledgets dipped into this preparation. Keep this in place by a cotton dressing held in place by a salolized gauze strip. Suppurating buboes are treated by irrigation with a strong solution of carbolized water and dressed with phenolized camphor. Buboes which are slow to suppurate are easily managed with injections of iodoform in ether. Inject one cubic centimetre three times a week by means of a hypodermatic syringe with a long needle.—*Lancet-Clinic.*

---

HEPATIC COLIC.—Lemoine uses the following methods in the treatment of hepatic colic. If no vomiting is present, he has recourse to ethereal solutions or those containing chloroform, as follows:

℞. Syrupi acaciæ, f℥iv.

Æther. sulphur., f℥i.

Or

℞. Chloroformi, ♏xv.

Tincturæ myrrh., ♏xv.

Mucilag. acaciæ, f℥ii.

Syrup., f℥iiss.

A tablespoonful of either of these prescriptions every fifteen minutes.—*Review Therapeutique.*

THE TREATMENT OF DIPHTHERIA.—The following plan is pursued by Dr. Alexander Fulton (*Medical News*, April 23): Application to the diphtheritic patches, by means of a throat brush, of a strong solution of nitrate of silver (forty grains to the ounce). If practicable this is followed by a gargle, such as,

℞. Tinct. kino............fl. ℥ij.

Glycerin...............fl. ʒij.

Ol. eucalyptol..........gtt. x.

M. Sig.—Teaspoonful in half ounce of water, as gargle.

Whether or not the gargle be used, the throat is dusted with the following:

℞. Hydrarg. chlor. corros....gr. i.
Pulv. sulphuris.............ʒi.

M. Sig.—Blow a "pinch" into throat every four hours (with insufflator).

From the commencement of the disease Dr. Fulton gives internally (according to age):

℞. Pulv. potass. chlorat...... ʒii.

Tr. ferri chloridi...........fl. ʒii.

Syr. limonis.............fl. ℥iii.

Ol. gualtheriæ..........gtt. iii.

M. Sig.—Teaspoonful every two or three hours.

A mixture of tincture of iodine and camphorated oil is applied externally over the site of the tonsil. This method of treatment "has been successful in thirty-seven consecutive cases."

---

SALOL FOR GONORRHŒA.—Dr. E. C. Underwood says that salol can reduce the duration of gon orrhœa to the lowest limits. This method consists in the regular employment of from forty to sixty grains of salol through the day. I order my patients to have four doses of from ten to fifteen grains each, taken immediately on rising in the morning, at 11 o'clock A. M., 4 o'clock P. M., and the last thing on retiring to bed at night. This is ordered in a powder or compressed tablets. Having known that many of these tablets passed through the intestinal canal without being absorbed, and in the form they were administered, I am now using the drug in the powder form. It is tasteless and is not complained of by patients. The dose is begun, unless this patient shows that the drug disagrees with him, with sixty grains a day, and continued until the discharge has become very meager Then it is gradually lessened. The author claims that better results follow this method than any other.—*West. Med. Reporter.*

SALICYLATE OF BISMUTH IN INFANTILE DIARRHŒAS.—In the *Meditzinskoie Obozrenie*, No. 6, 1892, p. 531, Dr. Mikhnevitch emphatically recommends the treatment of protracted diarrhœas in children under two years of age by the internal administration of salicylate of bismuth, after the formula:

℞. Bismuthi Salicylici, . . . . . . . . . gr. xxiv.

Gummi Arabici, . . . . . . . . . . ʒ i.

Sacchari albi, . . . . . . . . . . . ʒ iss.

Terendo adde—

Aquæ destillatæ, . . . . . . . . . ℥ ij.

Fiat lac. Tum adde—

Aquæ destillatæ, . . . . . . . . . ℥ vi.

M. D. S. To shake well before using. To give from one to two teaspoonfuls from three to six times a day.

Each teaspoonful of the mixture contains about one-half grain of the salicylate, which represents a normal individual dose (repeated three or four times daily) for an infant aged from six to eight months. The bottle should be kept in ice or cold water (to prevent nausea, sometime produced by the salicylate). In emaciated children the remedy, in largest doses, is apt to induce profuse perspiration, accompanied by general weakness. Hence, as soon as the sweating appears, the dose should be correspondingly diminished. In recent cases (of a few days' standing) the salicylate is useless.—*Ex.*

---

"GOLDEN GEMS OF GOODELL."—These recently enunciated, should be printed in large type and placed prominently before the eyes of every doctor in the sacred sanctum wherein he mostly pursues his studies. They are as follows, viz.:

First, always bear in mind "women have some organs outside of the pelvis."

Second, each neurotic case will usually have a tale of fret or grief, of cark and care, of wear and tear.

Third, scant or delayed or suppressed menstruation is far more frequently the result of nerve exhaustion than of uterine disease.

Fourth, anteflexion is not *per se* a pathological condition. It is so when associated with sterility or painful menstruation and only then does it need treatment.

Fifth, an irritable bladder is more often a nerve symptom than a uterine one.

Sixth, in a large number of cases of supposed or of actual uterine disease which displays marked gastric disturbance, if the tongue be clean the essential disease will be found to be neurotic, and must be treated as such.

Seventh, almost every supposed uterine case, characterized by excess of sensibility and by scantiness of will power, is essentially a neurosis.

Eighth, in the vast majority of cases in which a woman takes to bed and stays there indefinitely, from some supposed uterine lesion, she is bedridden from her brain, and not from her womb.

Lastly, uterine symptoms are not always present in cases of uterine disease, nor when present, even urgent, do they necessarily come from uterine disease, for they may be merely nerve counterfeits of uterine disease.

---

"IMPOTENCE" AND "STERILITY."—It is surprising how frequently the terms impotence and sterility are misused, notwithstanding the sharp and eminently proper distinction made between them—impotence denoting incapacity for copulating and sterility incapacity for fecundating. In a late book on Circumcision, widely reviewed, and showing on each page the author's intimate acquaintance with medical literature, it is sagely asserted that "Ultzmann observes that a man may be perfectly able to go through the procreative act, even to the great satisfaction of all parties concerned, and yet be *perfectly impotent*." What an anomalous state of affairs, indeed, that a man should be able to go through the procreative act, and yet be perfectly unable to do it, so to speak. In the *Journal of Cutaneous and Genito-Urinary Diseases*, for May, in quoting a paper by Dr. Finger, on the "Relationship between Gonorrhœal Inflammation in the Male and Sterility," the reporter, after evidently taking note of the difference in the causation of *potentia cœundi* and of *potentia generandi*, mixes them up again by explaining that *impotence* in the male is so frequent in connection with chronic posterior urethritis because of the chemical changes which the inflammation causes in the secretions of the part, rendering them noxious to the spermatozoa. Also, that "cicatricial contractions occurring about the openings of the ejaculatory ducts * * * may also give rise to *impotence*." Sterility is evidently the condition meant in both of these cases. [We take it for granted that an erring typo has made the reporter say that the "normal reaction of the prostatic fluid is acid."] There is no more reason for such indiscriminate use of these terms than there would be for calling typhus fever typhoid fever, or *vice versa*.—*Medical Fortnightly*.

---

TREATMENT OF ERYSIPELAS.—The treatment of Erysipelas which has yielded the best results in our experience (*Ther. Gaz.*) is one which we have already recommended elsewhere, and which in each and every case proves to be singularly efficient.

It consists in washing the part which is affected with a solution of bichloride of mercury, in the strength of 1 to 10,000, and then thoroughly anointing the skin with an ointment of ichthyol of the strength of two drachms of ichthyol to the ounce of lanolin or benzoated lard. This having been applied, the part is protected by a layer of salicylated cotton and a gauze covering ; or, if the disease involve the face, a piece of gauze can be used alone, with holes cut in it for the nose, eyes and mouth.

This line of local treatment in the early stages of the malady diminishes the pain and relieves the tension of the parts, at the same time seeming to limit the area involved. When the case is seen after the attack is well advanced, the same treatment rapidly reduces swelling and induration, and produces so rapid a change for the better that the patient soon recognizes the advances which he is making.

It is hardly necessary to add that full doses of tincture of chloride of iron should be given in such cases ; as much as 20 or 30 drops every four hours if the disease is severe. Since this has been written the article of Klim has been published, which gives practically the same treatment.—*Medical Review*.

MALARIA AND SURGICAL OPERATIONS.—After an investigation of the relation of malaria to surgical operations, Dr. Perez, Spain, concludes as follows (*Annals of Surgery*):

1. Before operating upon persons residing in malarial districts, although not presenting malarial symptoms, alway subject them to preliminary treatment by quinine, in order to avoid complications.

2. Individuals may be met with in whom there is a latent existence of the germs of malaria. These latter may develop when the strength of the patient has been lowered by hemorrhage, suppuration or other causes.

3. In deciding between a bloody and a bloodless method of operating select the latter, for beside avoiding hemorrhage, a mixed infection is also thus prevented.

4. If hemorrhage or intermittent pain follow the operation it may be met by various preparations of quinine.—*El Sigl. Medico.*

---

THE USE OF CHLORAL IN THE TREATMENT OF BOILS.—*Bull. Gen. de Therapeutique.* By M. Spehn.

The author recommends very highly as far superior to all other treatment the use of chloral externally in this troublesome class of affections. He directs that the boil be kept covered with a tampon of cotton-wool soaked in the following solution:

℞. Chloral hydrat....................................ʒiiss.

Aquæ,

Glycerin....................................aa fʒv.—M.

—*American Medical Magazine.*

---

ECHOES FROM THE MEDICAL COLLEGE EXAMINATIONS.—IN ANATOMY.—Prof. M.: "Mr. A., what structures pass through the foramen magnum?" Mr. A.: "The Aorta."

Prof. N.: "Mr. B., where does the duodenum begin?" Mr. B.: "At the mouth!"

---

THE best form of bismuth to use in the treatment of infantile diarrhœas is the salicylate, it being the most actively antiseptic.—*Medical World.*

